# Knock-In Reporter Mice Demonstrate that DNA Repair by Non-homologous End Joining Declines with Age

**DOI:** 10.1371/journal.pgen.1004511

**Published:** 2014-07-17

**Authors:** Amita Vaidya, Zhiyong Mao, Xiao Tian, Brianna Spencer, Andrei Seluanov, Vera Gorbunova

**Affiliations:** 1 Department of Biology, University of Rochester, Rochester, New York, United States of America; 2 School of Life Sciences and Technology, Tongji University, Yangpu district, Shanghai, China; Institute of Biotechnology, The University of Texas Health Science Center at San Antonio, United States of America

## Abstract

Accumulation of genome rearrangements is a characteristic of aged tissues. Since genome rearrangements result from faulty repair of DNA double strand breaks (DSBs), we hypothesized that DNA DSB repair becomes less efficient with age. The Non-Homologous End Joining (NHEJ) pathway repairs a majority of DSBs in vertebrates. To examine age-associated changes in NHEJ, we have generated an R26NHEJ mouse model in which a GFP-based NHEJ reporter cassette is knocked-in to the ROSA26 locus. In this model, NHEJ repair of DSBs generated by the site-specific endonuclease, I-SceI, reconstitutes a functional GFP gene. In this system NHEJ efficiency can be compared across tissues of the same mouse and in mice of different age. Using R26NHEJ mice, we found that NHEJ efficiency was higher in the skin, lung, and kidney fibroblasts, and lower in the heart fibroblasts and brain astrocytes. Furthermore, we observed that NHEJ efficiency declined with age. In the 24-month old animals compared to the 5-month old animals, NHEJ efficiency declined 1.8 to 3.8-fold, depending on the tissue, with the strongest decline observed in the skin fibroblasts. The sequence analysis of 300 independent NHEJ repair events showed that, regardless of age, mice utilize microhomology sequences at a significantly higher frequency than expected by chance. Furthermore, the frequency of microhomology-mediated end joining (MMEJ) events increased in the heart and lung fibroblasts of old mice, suggesting that NHEJ becomes more mutagenic with age. In summary, our study provides a versatile mouse model for the analysis of NHEJ in a wide range of tissues and demonstrates that DNA repair by NHEJ declines with age in mice, which could provide a mechanism for age-related genomic instability and increased cancer incidence with age.

## Author Summary

DNA damage disrupting both DNA strands, termed double strand breaks (DSBs), poses a threat to cell survival. If repaired inappropriately, such DNA breaks lead to genomic rearrangements, mutations, and ultimately cancer. Nonhomologous end joining (NHEJ) is the major pathway for repairing double-stranded breaks in mammals. Errors associated with NHEJ have been implicated in the aging process because mice with mutations in NHEJ genes exhibit premature aging. It remains unknown, however, whether NHEJ becomes impaired during normal aging. Studies of age-related changes in NHEJ have been hampered by the lack of a mouse model that would allow detection and quantification of NHEJ events. Here we report generation of NHEJ reporter mice containing a GFP-based NHEJ cassette knocked-into the ROSA26 locus. Using this mouse model, we were able to compare NHEJ across different tissues and demonstrate that NHEJ becomes less efficient and more error-prone with age. Our results provide a mechanism for age-related genomic instability and increased cancer incidence with age. The NHEJ reporter mice will be useful for a broad range of studies in the fields of aging and DNA repair.

## Introduction

The somatic mutation theory of aging posits that the accumulation of unrepaired somatic mutations over time leads to the ‘functional failure’ frequently observed during the course of aging [1]. Cellular DNA is a target of various endogenous and environmental insults, leading to DNA damage, of which double-stranded DNA breaks (DSBs) are the most damaging since they can lead to loss of genetic information via deletions or insertions and chromosomal rearrangements via translocations. Indeed, accumulation of such genome rearrangements have been observed in aged human and mouse tissues [2]–[8]. The appearance of genome rearrangements with age suggests that the process of DSB repair becomes compromised with age.

DSBs are repaired by two major pathways: non-homologous end joining (NHEJ) and homology-directed repair (HDR). NHEJ is faster than HDR [9] and does not require a homologous template, allowing it to take place in both dividing and non-dividing cells. As such, NHEJ is the primary DSB repair pathway in mammals. NHEJ is categorized as canonical NHEJ (c-NHEJ) and alternative NHEJ (alt-NHEJ), also called microhomology-mediated end joining (MMEJ) [10], [11]. c-NHEJ involves core component proteins, including Ku70, Ku80, DNA-PKcs, Artemis, and the Ligase IV complex (Lig IV, XRCC4, and XLF) [12]. NHEJ is also regulated by SIRT6 [13] and Werner syndrome proteins [14]. MMEJ is a DNA-PK-independent repair mechanism, which utilizes a distinct set of proteins, including but not limited to PARP-1, CtIP, XRCC1, and Ligase III [15]–[19]. MMEJ typically uses 5–25 bp microhomology for end-joining. MMEJ is inherently more mutagenic, leading to deletion of the sequences between microhomology regions [20].

The most compelling link between impaired NHEJ and aging is demonstrated by the appearance of accelerated aging in human patients and in mouse models with mutations in genes involved in NHEJ. For example, mice with loss-of-function mutations in Ku70, Ku80, and SIRT6 each exhibit symptoms of premature aging [21]–[25]. Werner syndrome patients display alopecia, atrophy, osteoporosis and die of cardiovascular disease or cancer at age 50 [26]. These mutant studies, however, do not tell us whether NHEJ is affected during normal aging.

Analysis of human lymphocytes and kidney, liver and skin cells from wild type mice showed an increase in the frequency of genome aberrations and mutations with age [2]–[8]. This accumulation of mutations and genomic rearrangements indicates a potential age-associated decline in the ability of somatic cells to repair DNA. Age-related decline in NHEJ in the brain has been suggested by *in vitro* plasmid rejoining assays in nuclear extracts [27], [28]. Furthermore, studies using human peripheral blood mononuclear cells have demonstrated reduced levels of Ku70 and Ku80 proteins [29]–[31], and a reduced capacity to repair radiation-induced breaks with age, as determined by comet assay [32]. Together, these studies suggest that the capacity to repair DSBs declines with age. However, studies analyzing the efficiency of the complete NHEJ reaction across tissues are missing. We previously measured NHEJ in replicatively senescent human fibroblasts and showed that NHEJ efficiency declines with replicative age [33], [34]. However, since chronological aging is more complex and heterogeneous than *in vitro* senescence, it remained to be determined whether NHEJ declines during organismal aging.

To study age-related changes in NHEJ, we generated the R26NHEJ mouse model, where a GFP-based reporter cassette is knocked-in to the ROSA26 locus. DSBs in the reporter cassette are generated by the expression of the I-SceI endonuclease. Repair of the breaks by NHEJ restores the functional GFP gene, which is then expressed from a constitutive ROSA26 promoter. The efficiency of NHEJ is quantified by the number of GFP^+^ cells. We then used this model to compare NHEJ across tissues and to conduct the analyses of age-associated changes in NHEJ repair in brain astrocytes and fibroblasts from heart, kidney, lung, and skin. We show that the efficiency of NHEJ declines 1.8 to 3.8-fold with age, depending on the tissue examined. In addition, heart and lung fibroblasts were found to utilize MMEJ more frequently with age. Our results provide evidence that NHEJ efficiency declines with age and reports a novel mouse model for *in vivo* studies of NHEJ.

## Results

### Generation of R26NHEJ reporter mice

**Figure 1:**
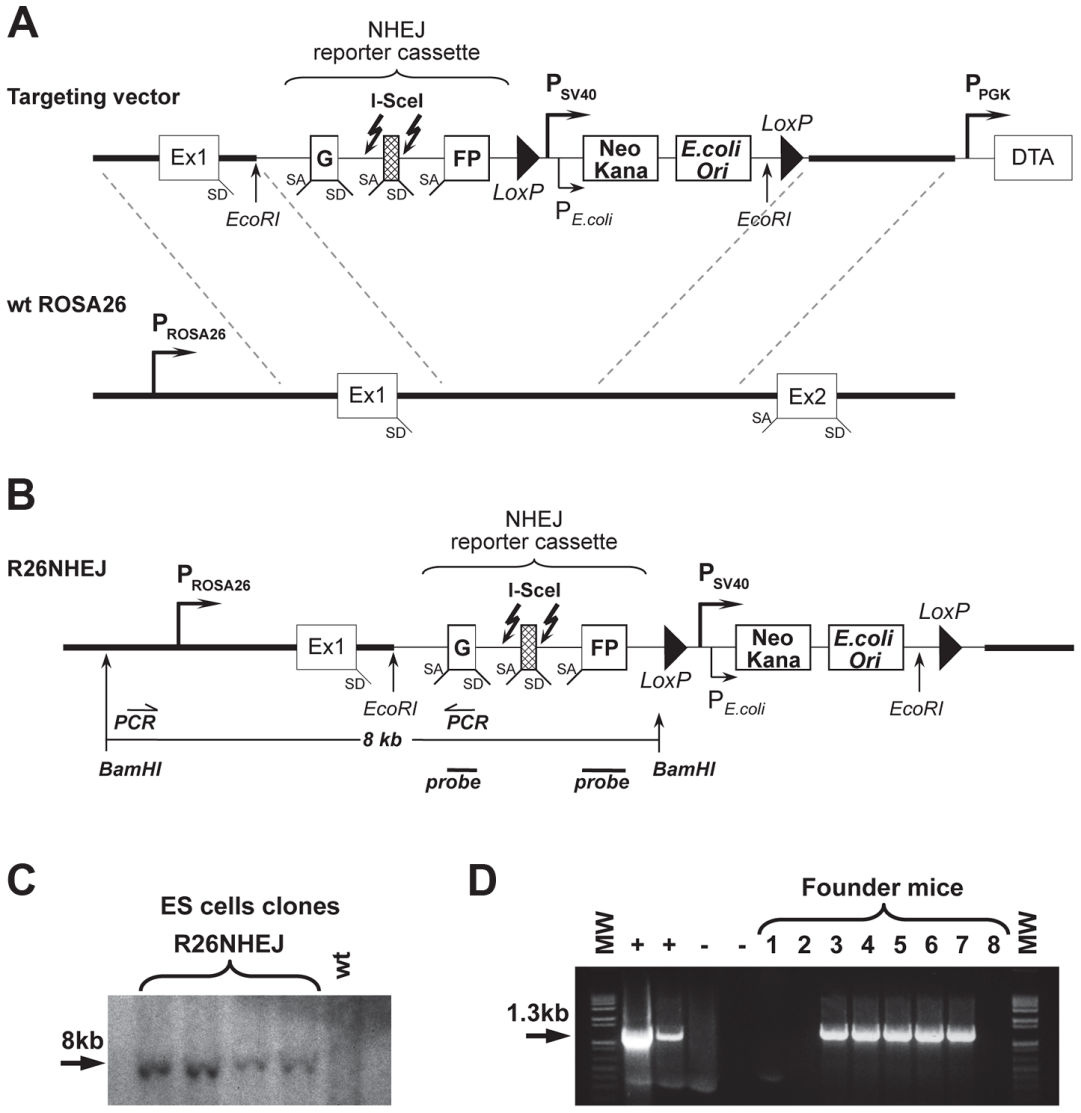
Generation of R26NHEJ knock-in mouse model. (**A**) The pROSA26PA-NHEJ vector containing NHEJ reporter construct targeted to the ROSA26 genomic sequence. The construct consists of GFP exons separated by the Pem1 intron, interrupted by the killer exon Ad2. Flanking Ad2 are unique I-SceI recognition sites for inducing DSB. Successful NHEJ repair leads to the reconstitution of GFP. Two *loxP* sites in direct orientation flanking Neomycin/Kanamycin genes (Neo/Kana) and Bacterial origin of Replication (OriC) are located downstream. This vector was targeted by homologous recombination into the endogenous ROSA26 locus of C57BL/6 mouse ES cells. (**B**) NHEJ construct integrated into ROSA26 locus in the mouse genome. (**C**) DNA from G418-resistant ES cell clones was digested with *Bam*HI and hybridized to a GFP probe (indicated in B). (**D**) Founder mice were genotyped using PCR primers indicated in (B). The positive control lanes contain PCR reactions with genomic DNA from ES cell clones shown in (C) as a template. Negative control lane contains PCR reactions with DNA from a wild-type C57BL/6 mouse as template and is followed by a No template control.

To study NHEJ, we generated the R26NHEJ mouse model where a GFP-based NHEJ reporter cassette was knocked-in to the ROSA26 locus. The NHEJ reporter cassette [35], [36] consists of the GFP gene interrupted by the Pem1 intron and an Ad exon flanked by I-SceI recognition sites (Figure 1A). The GFP gene in the NHEJ cassette is inactivated by the presence of the Ad exon. Upon induction of DSBs by I-SceI, the Ad exon is excised and rejoining of the intron sequences restores the GFP activity. The two I-SceI recognition sequences are in an inverted orientation such that the digestion of both sites generates non-compatible DNA ends (Table S1). NHEJ events can be quantified by the appearance of GFP^+^ cells. To generate the knock-in mice expressing the NHEJ reporter cassette under ROSA26 promoter, the NHEJ reporter was knocked into the ROSA26 locus following Exon 1. Gene targeting was performed in C57BL/6 mouse-derived embryonic stem (ES) cells (Figure 1B). We chose the C57BL/6 mouse strain because of its well-characterized aging pattern and relative longevity [37]. G418-resistant ES colonies were screened by Southern blot using *Bam*HI digestion (Figure 1C) and injected into mouse blastocysts. Chimeric males were obtained and mated with C57BL/6 females and the resulting offspring were genotyped using the GC-Rich PCR system (Figure 1D). Founder mice, confirmed positive for the targeted integration, were used to establish aging colonies of R26NHEJ mice.

### NHEJ efficiency declines with age

**Figure 2:**
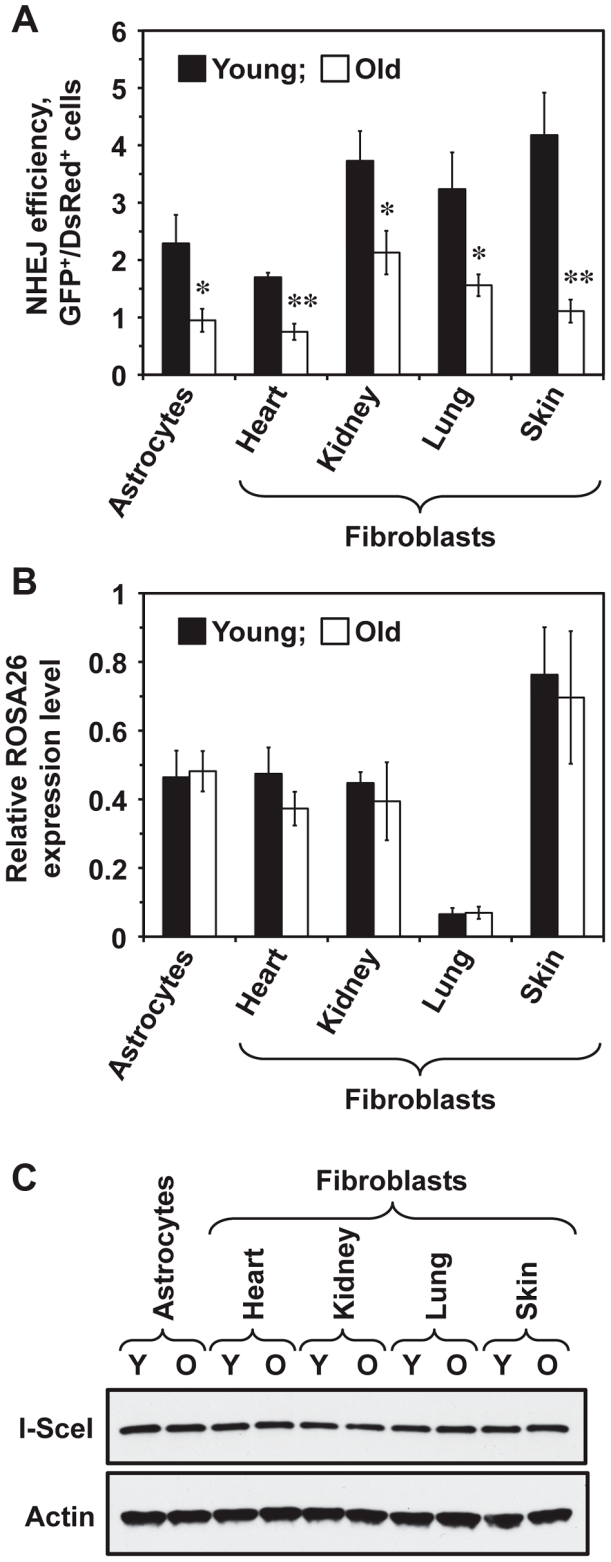
NHEJ efficiency declines with age in R26NHEJ mice. (**A**) Astrocytes and fibroblasts from heart, kidney, lung, and skin were isolated from 5 young and 5 old mice. After 2 passages, cells were transfected with 5 µg I-SceI for DSB induction and 0.025 µg DsRed to normalize the transfection efficiency. Three days later, cells were analyzed by FACS and NHEJ efficiency was calculated as the ratio of GFP^+^/DsRed^+^ cells. For each treatment, 20,000 cells were counted. At least 4 transfections were performed on cells from each mouse and the average NHEJ efficiency from young and old mice was plotted for the individual cell types analyzed. Error bars show s.e.m. (*p<0.05, **p<0.005, *t*-test). The data for individual mice is shown in Figure S2. (**B**) Transcription from ROSA26 locus containing the knocked-in NHEJ reporter does not change with age. Total RNA was extracted from the cells of young and old R26NHEJ mice. qRT-PCR was performed using primers homologous to the first exon of GFP and actin primers as the internal control. The experiment was repeated three times and error bars indicate s.d. (**C**) There is no significant difference in the I-SceI expression between cells from young and old mice. Total proteins were extracted from young and old fibroblasts and astrocytes and the I-SceI levels were analyzed by Western blot with antibodies to the HA-tag. β-actin was used as a loading control. The experiment was repeated three times and a representative image is shown.

To test whether NHEJ efficiency changes with age across multiple tissues, five young (5 month old) and five old (25 month old) heterozygous R26NHEJ mice were sacrificed to obtain brain astrocytes and fibroblasts from heart, kidneys, lungs, and skin. Primary cell isolates were characterized using astrocyte-specific GFAP (Glial Fibrillary Acidic Protein) and fibroblast-specific ER-TR7 markers to confirm the identity of cells (Figure S1). The number of cell passages prior to the NHEJ assay was kept at a minimum of 2–3 to avoid clonal selection. To measure NHEJ efficiency, we co-transfected the cells with I-SceI and DsRed plasmids and quantified the number of fluorescent cells by flow cytometry. NHEJ efficiency was calculated as the ratio of GFP^+^/DsRed^+^ cells to normalize for any differences in the transfection efficiency. The absence of GFP^+^ cells in mock-transfections indicated that the reporter construct was not leaky. We found that NHEJ efficiency varied across tissues (Figure 2A). In young mice, the NHEJ efficiency was higher in the kidney, lung, and skin fibroblasts and lower in the astrocytes and heart fibroblasts. Remarkably, we observed a significant, 1.8 to 3.8-fold, decline of NHEJ efficiency with age across all the tissues tested (Figure 2A). The age-related reduction of NHEJ efficiency was highest in the skin (3.8-fold).

### The observed decrease in NHEJ efficiency with age is not caused by reduced ROSA26 transcription or lower I-SceI expression

We chose the ROSA26 locus for integration of the NHEJ reporter cassette due to its ubiquitous expression and resistance to silencing [38]–[40]. However, whether the ROSA26 promoter is silenced with age had not been reported. To verify that the observed decrease in GFP repair events was not due to reduced ROSA26 transcription, we performed qRT-PCR on total RNA extracted from young and old cells, using primers annealing to Exon 1 of the ROSA26 locus and the first exon of the GFP ORF, to amplify the transcripts. ROSA26 transcription was found to remain unchanged with age in all the cell types tested (Figure 2B), indicating that the observed decrease in the number of GFP^+^ cells was not due to age-related changes in ROSA26 promoter expression. Although ROSA26 transcription was lower in the lung than in the other tissues, this did not correlate with the NHEJ efficiency because the FACS protocol used for the detection of NHEJ events scored the number of GFP^+^ cells and did not take into account the GFP intensity.

We next tested whether I-SceI expression changes with age. Western blot analysis of I-SceI levels 24 h after transfection did not show any appreciable differences in the I-SceI expression between young and old tissues or between cell types (Figure 2C), indicating that the observed reduction in NHEJ efficiency is not due to changes in I-SceI expression with age.

### NHEJ fidelity changes with age

To test whether the fidelity of NHEJ changes with age, we cloned and sequenced the NHEJ repair junctions. Genomic DNA was extracted from the cells and digested with *Pst*I to minimize the background of I-SceI uncut constructs. The NHEJ products were then amplified using primers that anneal within the Pem1 intron, cloned and sequenced (Figure S3). The primers we used allowed for the detection of deletions of up to 886 bp on the 5′ side and 750 bp on the 3′ side of the break. Multiple PCR reactions were set up in parallel, and only a single product from each reaction was included in the analysis to ensure that every original repair product was represented only once. A total of 300 sequences, 60 for each cell type were identified and are shown in Table S1. We observed NHEJ repair-associated deletions (ranging from 1 to 990 bp) and insertions (ranging from 1 to 138 bp) in different cell types.

**Figure 3:**
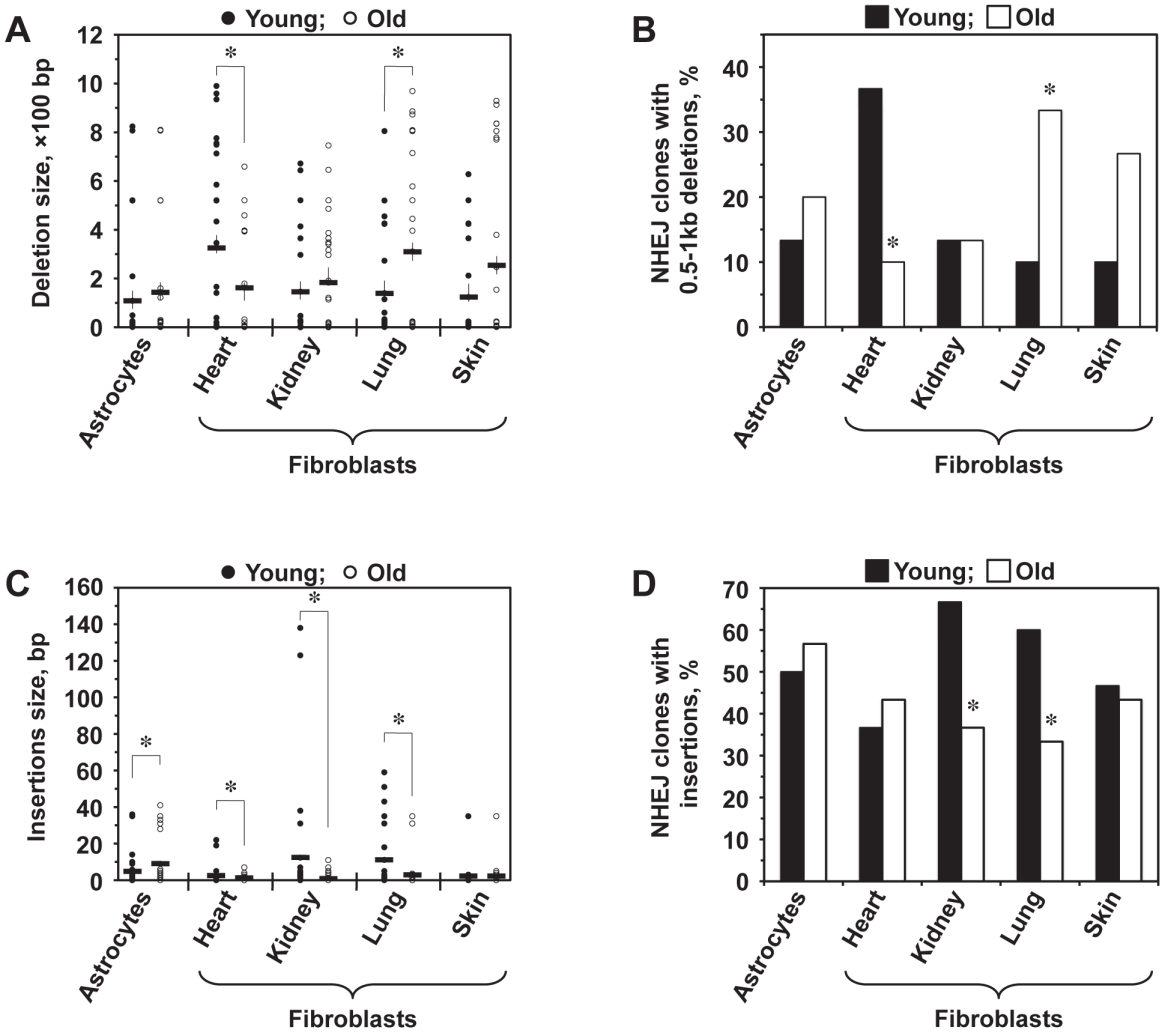
Analysis of deletions and insertions at NHEJ junctions in cells from young and old mice. A total of 300 independent junctions, 30 young and 30 old, for each cell type, were analyzed. The complete list of junction sequences is shown in Table S1. (**A**) Average deletion size decreases with age in heart fibroblasts and increases in lung and skin fibroblasts. Asterisk indicates significant difference between young and old mice (*p*<0.05, *t*-test). (**B**) Large deletions are more frequently found in old lung and skin fibroblasts and in young heart fibroblasts. The graph shows percentage of NHEJ clones containing deletions larger than 500 bp. Asterisk indicates significant difference between young and old mice (*p*<0.05, *t*-test). (**C**) Average size of insertions increases in astrocytes and decreases in kidney and lung fibroblasts. Asterisk indicates significant difference between young and old mice (*p*<0.05, *t*-test). (**D**) The frequency of insertions decreases with age in kidney and lung fibroblasts. Asterisk indicates significant difference between young and old mice (*p*<0.05, *t*-test).

Lung fibroblasts showed a significant increase in the average deletion size with age (*p*<0.05), and skin fibroblasts demonstrated a trend towards bigger deletions with age (*p*<0.1) (Figure 3A). Conversely, the average size of deletions decreased significantly (*p*<0.05) in old heart fibroblasts. In addition, for each cell type, deletion sizes were categorized into small (1–500 bp) and large (>500 bp). The frequency of NHEJ clones with large deletions was significantly increased in old lung fibroblasts and decreased in old heart fibroblasts (*p*<0.05) (Figure 3B). Astrocytes and kidney fibroblasts did not exhibit significant age-related changes with respect to deletion frequency or size.

A large number of junctions (33–67%) contained insertions, often combined with deletions. When we analyzed the average insertion sizes (Figure 3C), we found a significant increase in the insertion size with age in astrocytes (*p*<0.05). On the other hand, heart, kidney, and lung fibroblasts showed substantially smaller insertions with age. In addition, the frequency of clones with insertions was significantly greater in young kidney and lung fibroblasts compared to their older counterparts (*p*<0.05) (Figure 3D). No change with age in the insertion size and frequency was seen in skin fibroblasts. These data suggests that DNA synthesis leading to the generation of insertions is inhibited in old cells. In summary, NHEJ in aged cells exhibits a higher propensity to generate deletions and a reduced frequency of insertions.

### The frequency of MMEJ increases with age in heart and lung fibroblasts

**Figure 4:**
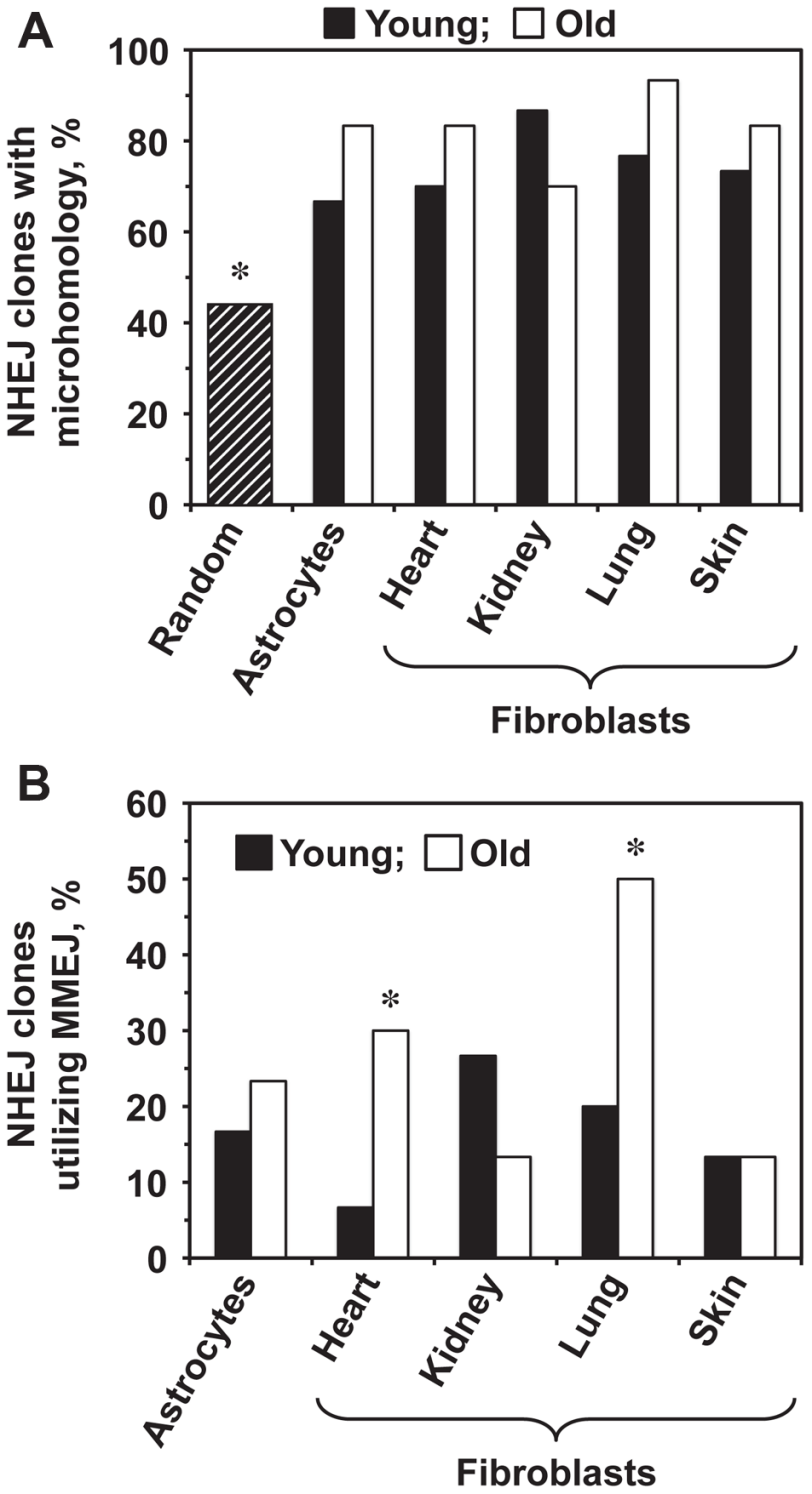
Old heart and lung fibroblasts show increased use of MMEJ. The complete list of junction sequences is shown in Table S1. (**A**) The sequenced repair junctions from both young and old cells contain more microhomologies than expected by chance. The hatched bar shows the theoretically calculated frequency of junctions with 1–16 nucleotides of microhomology (16 bp is the longest microhomology in Pem1 intron), if the ends were joined at random. Asterisk indicates that the observed frequency of microhomologies was significantly different from random (*p*<0.05, *t*-test). (**B**) Percentage of NHEJ junctions containing 5–16 bp of microhomology, classified as MMEJ events, increases with age in heart and lung fibroblasts. Asterisk indicates significant difference between junctions from young and old mice (*p*<0.05, *t*-test).

We next tested whether aging affects the choice of the NHEJ sub-pathway. The intron within the GFP gene contains multiple microhomology sequences ranging from 1–16 bp. The theoretical probability of obtaining a junction that contains a microhomology if the ends are joined at random within the Pem1 intron is 44% [35]. The percentage of sequenced repair junctions with microhomology was significantly greater than expected by chance (Figure 4A). Since the MMEJ pathway is distinguished by the presence of 5–25 bp microhomologies, we analyzed the frequencies of NHEJ repair clones containing microhomologies within this size range. The frequency of repair events utilizing microhomology increased significantly in the heart and lung fibroblasts and showed a trend towards increase in the astrocytes with age (Figure 4B). The use of microhomology did not change with age in the skin, and was reduced in the kidney (Figure 4B). In summary, these results suggest that the use of MMEJ pathway increases with age in several mouse tissues. It is possible that the increased utilization of MMEJ is a compensatory mechanism to cope with the decline in the c-NHEJ function. Since MMEJ is a more error-prone pathway, this change in the pathway use may lead to a further increase of the mutation load by increasing the size and frequency of deletion events.

## Discussion

Aged tissues accumulate genomic rearrangements [41] and defects in DSB repair have been implicated in the aging process [42]–[44]. Here, we tested whether the process of NHEJ deteriorates during normal aging in the mouse. To address this question, we generated the R26NHEJ mouse model, in which a GFP-based NHEJ reporter cassette was knocked-in downstream of the ROSA26 promoter. To our knowledge, this is the first instance of a mouse model that can quantify NHEJ repair in multiple mouse tissues. The advantage of this model is that the NHEJ reporter counts completed repair events, rather than intermediate steps such as formation of γH2A.X foci or recruitment of DNA repair proteins. Furthermore, only the NHEJ events are scored, offering an advantage over comet assays, which cannot distinguish between DSB repair pathways. We found no age-related changes in the expression of the ROSA26 promoter (Figure 2), making this locus an ideal targeting site for aging studies. This is consistent with the fact that ROSA26 promoter has an open chromatin configuration and virtually no detectable methylation of CpG islands [45]. Another unique feature of the R26NHEJ mouse is that NHEJ events can be analyzed in the same chromosomal location across multiple cell and tissue types. ROSA26 expression was lower in the lung fibroblasts than in the other cell types, which may reflect the chromatin state of this locus in the lung. However, because our FACS parameters were set to include the majority of GFP^+^ cells, regardless of the fluorescence intensity, lower ROSA26 expression did not result in lower detected NHEJ efficiency in lung fibroblasts relative to the other tissues. This shows that the R26NHEJ system is suitable for comparison of NHEJ across tissues.

NHEJ repair events can be detected *in vivo* using this system by expressing I-SceI in the mouse to induce DSBs. Several approaches to induce DSBs *in vivo* were attempted including delivery of I-SceI endonuclease by adenoviral vector, adeno-associated (AAV) vectors, nanoparticles, or hydrodynamic tail-vein injections. The best results were obtained with an AAV9 vector but the frequency of the events was not sufficiently robust for quantitative analysis of repair events with reliable statistics. Therefore, we chose to examine the NHEJ efficiency *ex vivo* in freshly isolated fibroblasts from the heart, kidney, lung, skin, and brain astrocytes. We found that NHEJ efficiency declined significantly in all the cell types tested. The decline in repair efficiency ranged from 1.8 to 3.8-fold, with the strongest 3.8-fold decline observed in skin fibroblasts. Inefficient NHEJ can impede the cell cycle or lead to cell death. Furthermore, inefficient NHEJ may leave broken DNA ends exposed for a longer time, facilitating the formation of inappropriate junctions and large-scale genomic rearrangements previously observed in aged tissues [6], [8]. Indeed, we observed an increase in the deletion sizes (Figure 3A, B) with age. In addition to promoting tumorigenesis, such rearrangements could disrupt higher order chromatin structure and contribute to age-related dysregulation of gene expression patterns [46].

Fibroblasts and astrocytes play important roles in maintaining the tissue structure and function [47], [48]. Fibroblasts primarily form the extracellular matrix around cells, playing important roles in the structure, function, repair, signaling, and death of the surrounding specialized cells [49]. Astrocytes or glial cells are essential for providing structural, metabolic, and functional support to neurons as well as for memory formation, DNA repair, and scarring during trauma [50]. Impaired function of these cells, due to inefficient NHEJ, could contribute to age-related decline in the tissue function. Although the current study was limited to fibroblasts and astrocytes, the R26NHEJ mouse can be utilized to study NHEJ in all cell types, provided efficient delivery of the I-SceI endonuclease. As mice frequently develop lymphomas with age, it would be interesting to examine NHEJ in their hematopoietic tissues. We are currently generating a lentiviral vector to express I-SceI in hematopoietic cells.

The R26NHEJ mouse has allowed us to compare NHEJ efficiency in the same genomic locus across different tissues and different ages. We found that NHEJ is more efficient in the kidney, lung, and skin fibroblasts than in astrocytes and heart fibroblasts. The strongest decline in NHEJ with age was observed in the fibroblasts from two epithelial tissues, skin and lung. This could be explained by the higher number of cell divisions that occur over time in these tissue compartments, leading to faster cell aging and increased genomic instability. Understanding tissue specific differences in DNA repair pathways can shed light on the tissue specificity of familial cancer-predisposing mutations in genes such as BRCA1, BRCA2, or APC.

The Pem1 intron of the R26NHEJ construct can tolerate sizeable deletions and insertions, enabling the analysis of a wide range of NHEJ events at the repair junctions. Repair-associated aberrations exhibited great variation at different ages and in different tissues. Notably, NHEJ events in old lung and skin fibroblasts were associated with larger deletions, while NHEJ events in old heart fibroblasts were associated with smaller deletions. Large deletions could be a direct result of less efficient NHEJ resulting in broken ends being exposed to nucleases for a longer time. In addition to deletions, 33–67% of the junctions contained insertions. The insertion size increased with age in the astrocytes but decreased in the heart, kidney, and lung fibroblasts. The frequency of clones with insertions also decreased in the kidney and lung fibroblasts. Since insertion formation requires DNA synthesis, this decrease could be explained by a decrease in repair-associated DNA synthesis in old mice. We also found a considerable increase in MMEJ events in old heart and lung fibroblasts. The increased propensity for MMEJ suggests a possible switch from c-NHEJ to MMEJ, rendering the end-joining process more mutagenic, leading to genomic instability. Interestingly, compromised NHEJ has been implicated in tumorigenesis with an observed increase in alt-NHEJ mechanisms. Human urinary bladder carcinomas and myeloid leukemia exhibit high levels of MMEJ [51], [52]. As cancer incidence increases exponentially with age, the shift from c-NHEJ towards MMEJ observed in the heart and lung fibroblasts, may contribute to this process.

We previously examined the changes in NHEJ during replicative senescence of human fibroblasts [35]. Similar to the current study, we observed up to 4-fold decline in repair efficiency during replicative aging. Aged tissues contain a complex mixture of cells, including replicatively senescent cells, cells senescent due to stress, and proliferating cells; therefore, it was important to test whether NHEJ declines in aged tissues. Our findings, based on the NHEJ reporter mice, demonstrate that repair efficiency declines in aged tissues. Interestingly, the effect of replicative senescence and aging on the ratio of c-NHEJ to MMEJ was different between human and mouse cells. Although repair junctions from replicatively senescent human cells contained larger deletions, the use of microhomologies was markedly reduced [35]. On the other hand, MMEJ frequency was found to increase with age in mice. This difference may contribute to the higher genome stability and lower cancer incidence in humans compared to mice.

In conclusion, we show that NHEJ becomes less efficient with age, which may contribute to increased genomic instability and cancer incidence. The ubiquitously expressed, chromosomal NHEJ construct makes the R26NHEJ mouse a powerful tool for the analysis of NHEJ. These mice can be bred with various mice mutated for genes involved in DNA repair and aging. Furthermore, the effect of pharmacological and dietary interventions on NHEJ could be analyzed. These mice can also be used to compare NHEJ between different tissues and cell types, which could shed light on the tissue-specific differences in tumor susceptibility.

## Methods

### Ethics statement

All mouse experiments were performed in accordance with the guidelines established by the University of Rochester Committee on Animal Resources (UCAR). All the experimental protocols were approved by UCAR and the approval number is 101423. Mice were euthanized by CO_2_ inhalation according to the approved protocol.

### Mice

The NHEJ reporter cassette was inserted into the Multiple Cloning Site of the pBigT vector [40] and this entire construct was cloned into the pROSA26PA plasmid between the arms of the ROSA26 genomic sequence [39]. This pROSA26PA-NHEJ vector was then targeted into C57BL/6 mouse-derived embryonic stem (ES) cells. The transfection of ES cells and subsequent blastocyst injections were performed by inGenious Targeting Laboratory. We chose the C57BL/6 mouse strain because of its well-characterized aging pattern and relative longevity [37]. G418-resistant ES colonies were screened by Southern blot using *Bam*HI digestion. ES cell clones that showed the desired chromosomal integration were injected into blastocysts, which were then transplanted into pseudopregnant female mice. Chimeric males obtained were mated with C57BL/6 females and the resulting 8 offspring were genotyped. Five out of eight founder mice confirmed positive for the targeted integration were then used to establish aging colonies of R26NHEJ mice.

R26NHEJ founder mice were genotyped using a GC-Rich PCR System (Roche) with the Forward 5′ primer 5′- CGGGACTCTGGCGGGAGGGCGGCTTGGTGC - 3′ binding to the ROSA26 promoter sequence involved in homologous recombination and the Reverse 3′ primer 5′- GTTCTAGAGCGGCCTCGACTCTACGATACC - 3′ binding to the internal sequence of the ROSA26NHEJ construct to selectively amplify a 1.3 kb band.

To distinguish between heterozygous and homozygous R26NHEJ mice, another primer pair was used in conjunction with the above PCR genotyping primers. The Forward Chr6-Sens primer 5′- AGTCGCTCTGAGTTGTTATCAGTAAGG - 3′ is homologous to the Chromosome 6 sequence upstream of the ROSA26 locus and the Reverse Chr6-Anti primer 5′- GGTTTCATGAGTCATCAGACTTCTAAGATCAGG - 3′ can bind only to the Chromosome 6 sequence, past the ROSA26 locus. Consequently, wild-type C57BL/6 (+/+) and heterozygous (N/+) mice with 1 copy of the NHEJ construct can amplify the 741 bp band with these primers while homozygous (N/N) mice with 2 copies of the NHEJ construct cannot.

### Cell culture

Primary cultures were established from brain astrocytes and fibroblasts from heart, kidney, lungs, and skin of R26NHEJ mice. Briefly, mouse brains were mechanically digested with 0.25% Trypsin/EDTA in PBS for 10 min, washed, and plated on Poly-l-lysine- (Sigma) coated plates. Seven to ten days post-isolation, supernatant and debris were aspirated while confluent astrocytes remained adhered to the plates. Fibroblasts were isolated using protocols described previously [53]. All the cells were grown using DMEM/F12 media (Gibco) supplemented with 10% Fetal Bovine Serum (Gibco), and 1% Penicillin/Streptomycin (Gibco) under conditions of 3% O_2_, 5% CO_2_ at 37°C.

### Transfections

Fibroblasts were transfected with 5 µg pCMV-I-SceI for DSB generation and 0.025 µg pCMV-DsRed2 plasmid to normalize for transfection efficiency using program U-023 on Amaxa Nucleofector II using NHDF solution (Lonza). Similarly, astrocytes were transfected using program T-020 and PMGC solution (Lonza). Three days post-transfection, samples were analyzed for GFP^+^ and DsRed^+^ cells using Fluorescence Associated cell sorting (Canto II). Multiple transfections were performed for individual cell lines and NHEJ efficiency was calculated as the ratio of GFP^+^ to DsRed^+^ cells.

### Immunofluorescence

Mouse astrocytes and skin fibroblasts were seeded on slides, grown for 2 days, and fixed using 4% paraformaldehyde. Cells were permeabilized with 0.25% Triton X-100, washed with PBS, and blocked with 1% BSA for 1 h. Astrocytes and fibroblasts were incubated with the primary antibodies, anti-Glial Fibrillary Acidic Protein (GFAP) antibody (Abcam; ab7260) and ER-TR7 Fibroblast-specific antibody (MA1-40076; Thermo Scientific Pierce;) respectively, for 16 h at 4°C. After washing with PBS, the secondary antibodies, goat-anti-rabbit-FITC (Abcam; ab6717) and goat-anti-rat-Cy5 (Abcam; ab6955), respectively, were used, followed by 1 µg/mL DAPI staining for 2 min. Vectashield Mounting Medium (Vector Laboratory; H-1000) was added to the slides along with coverslip and cells were imaged on Leica Confocal Microscope.

### Quantitative RT-PCR

Total RNA was extracted from astrocytes and fibroblasts using RNeasy Mini Kit (Qiagen). Following DNAse treatment, RNA was incubated with Oligo(dT) 18 primer and SuperScript III Reverse Transcriptase (Invitrogen) to produce cDNA. cDNA dilutions ranging from 1, 1/2, 1/4, 1/8, and 1/16 were set up to create standard curves. RT-PCR was performed using FastStart Universal SYBR Green Master (Roche) and control Actin primers (Ambion). Primers used for cDNA amplification were: GFP-Ex1 Forward, 5′- CTCGCGGTTGAGGACAAACTCTTCGCGGTCTTTCCAGTGGGG-3′ and GFP-Ex1 Reverse, 5′- GACTTGAAGAAGTCGTGCTGCTTCATGTGGTCGGGGTAGCGGCTGA-3′.

### Western blot for I-SceI expression

Equal number of cells (1×10^6^) isolated from young and old mice were transfected with 5 µg of pCMV-I-SceI plasmid. Twenty four hours after transfection, SDS-PAGE was performed and I-SceI was detected using anti-HA antibody (Santa Cruz; sc-7392). β-Actin (Santa Cruz; sc-47778) was used as the loading control.

### Sequencing of NHEJ clones

Genomic DNA was extracted from astrocytes and fibroblasts transfected with pCMV-ISceI and pCMV-DsRed2 plasmids, using the phenol-chloroform method. DSB repair sites in the NHEJ construct were amplified by PCR and GC-PCR using various primer combinations; PEM1 Forward, 5′-GCTAAGTGCTTAGTAAAGCAATAGACTGCAT-3′, 5′- GGCTACCTCCAGTTCTAAGGCTGCACTCCA-3′ and PEM1 Reverse, 5′- CTAGGTACTAGGAATTGAACCTAGG-3′, 5′- GGACTAGTAATTGTTTAACATGTGGGAAGTT-3′. Amplified DNA was electrophoresed in 1% Agarose gel and purified using QIAquick Gel Extraction Kit (Qiagen). These NHEJ fragments were cloned into a TA vector using the TOPO TA cloning kit (Invitrogen) and sent for sequencing. Sequenced TA-NHEJ clones were aligned and analyzed using the SerialCloner software.

### Statistical analyses

Significance analysis for the percentage of deletions, insertions, and microhomologies was calculated using two sample *t*-test between percents using StatPac calculator. In all other cases, significance analysis was calculated using Student's unpaired *t*-test on GraphPad software.

## Supporting Information

Figure S1 Identification of primary cells isolated from R26NHEJ mice. Immunofluorescence staining of (**A**) astrocytes and (**B**) skin fibroblasts derived from R26NHEJ mice. FITC-labeled astrocyte-specific anti-GFAP antibody and Cy-5-labeled fibroblast-specific ER-TR7 antibody was used to confirm the purity of primary cultures. DAPI was used as the nuclear stain.
(EPS)

Figure S2 NHEJ efficiency of individual R26NHEJ mice. Astrocytes and fibroblasts from heart, kidney, lung, and skin were isolated from 5 young and 5 old mice. After two passages, cells were transfected with 5 µg I-SceI for DSB induction and 0.025 µg DsRed to normalize the transfection efficiency. Three days later, cells were analyzed by FACS and NHEJ efficiency was calculated as the ratio of GFP^+^/DsRed^+^ cells. At least four transfections were performed on cells from each mouse and the individual bars denote average NHEJ efficiency for each mouse. Error bars show s.d. Darker and corresponding lighter bars indicate young and old mice, respectively.
(EPS)

Figure S3 Cloning strategy for rescue and analysis of NHEJ repair junctions. The diagram shows GFP exons separated by the Pem1 intron and Ad exon flanked by I-SceI sites (not drawn to scale). Following I-SceI transfections and NHEJ assay, genomic DNA was extracted from cells and digested with *Pst*I to eliminate reporter cassettes that were not cut by I-SceI. Genomic DNA was then amplified with the indicated primers that are homologous to the Pem1 intron sequence. PCR products were cloned into the TOPO vector for sequencing. Clones were then aligned to the original NHEJ construct to analyze aberrations at repair junctions. To avoid counting identical PCR products as independent events, multiple PCR reactions were set up using genomic DNA from each NHEJ repair assay, and only one junction with a unique sequence from each PCR reaction was included in the subsequent analysis.
(EPS)

Table S1 Sequences of NHEJ repair junctions in astrocytes and fibroblasts of young and old R26NHEJ mice.
(DOCX)
