# Supplementary material for: Knock-In Reporter Mice Demonstrate that DNA Repair by Non-homologous End Joining Declines with Age
Source: PLoS Genet. 2014 Jul 17;10(7):e1004511. doi: 10.1371/journal.pgen.1004511 (PMC4102425; doi:10.1371/journal.pgen.1004511)
Supplement: Table S1 — Sequences of NHEJ repair junctions in astrocytes and fibroblasts of young and old R26NHEJ mice. (DOCX) [file pgen.1004511.s004.docx]

**Table S1.** Sequences of NHEJ repair junctions in astrocytes and fibroblasts of young and old R26NHEJ mice.

**I-SceI-cut generating incompatible ends^a^**

**-----------------TAGGGATAA CCCTA------------------**

**-----------------ATCCC AATAGGGAT------------------**

**# Repaired junctions D.I.M^b^**

**Young Brain Astrocytes**

**ATGGGTGTTTCCAAGAAGCTTTAGGGATAA CCCTAAAGCTTGTCACAGACCCCTCCTGCT cut ends**

**1 ATGGGTGTTTCCAAGAAGCTTTAGGGATA TCCCTAAAGCTTGTCACAGACCCCTCCTGCT 1.1.2**

**2 ATGGGTGTTTCCAAGAAGCTTTAGGGAT CCCTAAAGCTTGTCACAGACCCCTCCTGCT 2.0.2**

**3 ATGGGTGTTTCCAAGAAGCTTTAGGGAT TATCCCTAAAGCTTGTCACAGACCCCTCCTGCT 2.3.1**

**4 ATGGGTGTTTCCAAGAAGCTTTAGGGAT TATCCCTAAAGCTTGTCACAGACCCCTCCTGCT 2.3.1**

**5 ATGGGTGTTTCCAAGAAGCTTTAGGG CCCTAAAGCTTGTCACAGACCCCTCCTGCT 4.0.0**

**6 ATGGGTGTTTCCAAGAAGCTTTAGGG ATCCCTAAAGCTTGTCACAGACCCCTCCTGCT 4.2.2**

**7 ATGGGTGTTTCCAAGAAGCTTTAAGGG ATCCCTAAAGCTTGTCACAGACCCCTCCTGCT 4.3.2**

**8 ATGGGTGTTTCCAAGAAGCTTTAGGG TTATCCCTAAAGCTTGTCACAGACCCCTCCTGCT 4.4.4**

**9 ATGGGTGTTTCCAAGAAGCTTTAGGG AAAGCTTGTCACAGACCCCTCCTGCT 9.0.0**

**10 ATGGGTGTTTCCAAGA (31)CGCGTGTCACAGACCCCTCCTGCT 14.35.4**

**11 ATGGGTGTTTCCAAGAAGCTTTAGGGAGGCTAGAGT CAGACCCCTCCTGCT 18.10.0**

**12 ATGGGTGTTTCCAAGAAGCTTTAGGGAGGCTAGAGT CAGACCCCTCCTGCT 18.10.0**

**13 ATGGGTGTTTCCAAGAAGCTTTAGGG CAGACCCCTCCTGCT 19.0.0**

**14 ATGGGTGTTTCCAAGAAGCTTTAGGG CAGACCCCTCCTGCT 19.0.0**

**15 ATGGGTGTTTCCAAGAAGCTTTAGGG CAGACCCCTCCTGCT 19.0.0**

**16 ATGGGTGTTTCCAAGAAGCTT GTCACAGACCCCTCCTGCT 20.0.6**

**17 ATGGGTGTTTCCAAGAAGCTT GTCACAGACCCCTCCTGCT 20.0.6**

**18 ATGGGTGTTTCCAAGAAGCTT GTCACAGACCCCTCCTGCT 20.0.6**

**19 ATGGGTGTTTCCAAGAAGCTT GTCACAGACCCCTCCTGCT 20.0.6**

**20 ATGGGTGTTTCCAAGAAGCTT GTCACAGACCCCTCCTGCT 20.0.6**

**21 ATGGGTGTTTCCAACAGGCTTCTCACTT ACCCCTCCTGCT 20.9.1**

**22 ATGGGTGTTTCCGGAAAGCTTGTCCTT ACCCCTCCTGCT 22.14.0**

**23 ATGGGTGTTTCCAAGACGCG(31) TGTCACAGACCCCTCCTGCT 24.35.4**

**24 ATGGGTGTTTCCAAGGCG(33) TGTCACAGACCCCTCCTGCT 25.36.3**

**25 ATTC AAGCTTGTCACAGACCCCTCCTGCT 49.0.0**

**26 CTTC AATG 209.0.1**

**27 CAGAT CCA 520.0.0**

**28 AAGA AAC 521.0.2**

**29 AAG AGAC 807.2.2**

**30 AGAT TGTCACT 824.5.1**

**Old Brain Astrocytes**

**ATGGGTGTTTCCAAGAAGCTTTAGGGATAA CCCTAAAGCTTGTCACAGACCCCTCCTGCT cut ends**

**31 ATGGGTGTTTCCAAGAAGCTTTAGGGATA TCCCTAAAGCTTGTCACAGACCCCTCCTGCT 1.1.2**

**32 ATGGGTGTTTCCAAGAAGCTTTAGGGATA TCCCTAAAGCTTGTCACAGACCCCTCCTGCT 1.1.2**

**33 ATGGGTGTTTCCAAGAAGCTTTAGGGATAAC CCTAAAGCTTGTCACAGACCCCTCCTGCT 1.1.4**

**34 ATGGGTGTTTCCAAGAAGCTTTAGGGATAACAG CCTAAAGCTTGTCACAGACCCCTCCTGCT 1.3.0**

**35 ATGGGTGTTTCCAAGAAGCTTTAGG AATTATCCCTAAAGCTTGTCACAGACCCCTCCTGCT 3.6.1**

**36 ATGGGTGTTTCCAAGAAGCTTTAGGG TTATCCCTAAAGCTTGTCACAGACCCCTCCTGCT 4.4.4**

**37 ATGGGTGTTTCCAAGAAGCTTTAGGGATA AAAGCTTGTCACAGACCCCTCCTGCT 5.0.1**

**38 ATGGGTGTTTCCAAGAAGCTT GTCACAGACCCCTCCTGCT 20.0.6**

**39 ATGGGTGTTTCCAAGAAGCTT GTCACAGACCCCTCCTGCT 20.0.6**

**40 ATGGGTGTTTCCAAGAAGCTT GTCACAGACCCCTCCTGCT 20.0.6**

**41 ATGGGTGTTTCCAAGAAGCTT GTCACAGACCCCTCCTGCT 20.0.6**

**42 ATGGGTGTTTCCAAGAAGCTT GTCACAGACCCCTCCTGCT 20.0.6**

**43 ATGGGTGTTTCCAAGAAGCTT GTCACAGACCCCTCCTGCT 20.0.6**

**44 ATGGGTGTTTCCAAGAA(28) GCTTGTCACAGACCCCTCCTGCT 20.28.2**

**45 ATGGGTGTTTCCAAGAC(30) GCTTGTCACAGACCCCTCCTGCT 21.31.1**

**46 TGGGTGTTTCCAAGAAGCTT GGNACAGACCCCTCCTGCT 23.3.6**

**47 ATGGGTGTTTCCAAGA (33)TGTCACAGACCCCTCCTGCT 23.33.0**

**48 ATGGGTGTTTCCAAGACGCG(31) TGTCACAGACCCCTCCTGCT 24.35.4**

**49 ATGGGTGTTTCCAAGACGCG(31) TGTCACAGACCCCTCCTGCT 24.35.4**

**50 ATGGGTGTTTCCAAGACGCG(31) TGTCACAGACCCCTCCTGCT 24.35.4**

**51 ATGGGTGTTTCCAAGAAGCTT GTCNNAGACCCCTCCTGCT 25.5.0**

**52 ATGGGTGTTTCCAAGA (41)AGACCCCTCCTGCT 30.41.0**

**53 CCTTGG AAAGCTTGTCACAGACCCCTCCTGCT 122.0.0**

**54 CTGA CACAGACCCCTCCTGCT 160.0.2**

**55 CAGAT CCA 520.0.0**

**56 AAGA AAC 521.0.2**

**57 AAGA AAC 521.0.2**

**58 AAGA AAC 521.0.2**

**59 AAG AGAC 807.2.2**

**60 AAG AGAGTC 810.5.2**

**Young Heart Fibroblasts**

**ATGGGTGTTTCCAAGAAGCTTTAGGGATAA CCCTAAAGCTTGTCACAGACCCCTCCTGCT cut ends**

**61 ATGGGTGTTTCCAAGAAGCTTTAGGGATA TCCCTAAAGCTTGTCACAGACCCCTCCTGCT 1.1.2**

**62 ATGGGTGTTTCCAAGAAGCTTTAGGGATAAC CCTAAAGCTTGTCACAGACCCCTCCTGCT 1.1.4**

**63 ATGGGTGTTTCCAAGAAGCTTTAGGGATA ATCCTAAAGCTTGTCACAGACCCCTCCTGCT 1.2.1**

**64 ATGGGTGTTTCCAAGAAGCTTTAGGGAT CCCTAAAGCTTGTCACAGACCCCTCCTGCT 2.0.2**

**65 ATGGGTGTTTCCAAGAAGCTTTAGGGAT CCCTAAAGCTTGTCACAGACCCCTCCTGCT 2.0.2**

**66 ATGGGTGTTTCCAAGAAGCTTTAGGGAT CCCTAAAGCTTGTCACAGACCCCTCCTGCT 2.0.2**

**67 ATGGGTGTTTCCAAGAAGCTTTAGGGAT TATCCCTAAAGCTTGTCACAGACCCCTCCTGCT 2.3.1**

**68 ATGGGTGTTTCCAAGAAGCTTTAGGGAT NATCCCTAAAGCTTGTCACAGACCCCTCCTGCT 2.3.2**

**69 ATGGGTGTTTCCAAGAAGCTTTAGGG ATCCCTAAAGCTTGTCACAGACCCCTCCTGCT 4.2.2**

**70 ATGGGTGTTTCCAAGAAGCTTTAGG GTTATCCCTAAAGCTTGTCACAGACCCCTCCTGCT 5.5.4**

**71 ATGGGTGTTTCCAAGAAGCTTT CCCTAAAGCTTGTCACAGACCCCTCCTGCT 8.0.1**

**72 ATGGGTGTTTCCAAGAAGCTT GTCACAGACCCCTCCTGCT 20.0.6**

**73 ATGGGTGTTTCCAAGAAGCTT GTCACAGACCCCTCCTGCT 20.0.6**

**74 ATGGGTG AGACCCCTCCTGCT 39.0.0**

**75 CCCACA ACTTG 141.0.2**

**76 ATGGGTGTTTCCAAGAAGCTTTAGGGATAA(16)TCT TTGCA 166.19.1**

**77 GGGT AGGA 346.0.0**

**78 GGGT AGGA 346.0.0**

**79 GATCC TCCTG 434.0.2**

**80 CAGAT CCA 520.0.0**

**81 AAGA AAC 521.0.2**

**82 CAAGCA GGG 585.0.4**

**83 CAC TGT 713.0.0**

**84 CATG GGCC 749.0.1**

**85 GCAA TTAC 754.0.3**

**86 TTGC GATTA 755.1.0**

**87 CCC(22) CAA 776.22.0**

**88 GAGGG CCT 934.0.4**

**89 GAT AGT 959.0.0**

**90 AGCA GGAGGC 990.5.0**

**Old Heart Fibroblasts**

**ATGGGTGTTTCCAAGAAGCTTTAGGGATAA CCCTAAAGCTTGTCACAGACCCCTCCTGCT cut ends**

**91 ATGGGTGTTTCCAAGAAGCTTTAGGGATA TCCCTAAAGCTTGTCACAGACCCCTCCTGCT 1.1.1**

**92 ATGGGTGTTTCCAAGAAGCTTTAGGGATA TCCCTAAAGCTTGTCACAGACCCCTCCTGCT 1.1.2**

**93 ATGGGTGTTTCCAAGAAGCTTTAGGGATA TCCCTAAAGCTTGTCACAGACCCCTCCTGCT 1.1.2**

**94 ATGGGTGTTTCCAAGAAGCTTTAGGGAT CCCTAAAGCTTGTCACAGACCCCTCCTGCT 2.0.2**

**95 ATGGGTGTTTCCAAGAAGCTTTAGGGAT CCCTAAAGCTTGTCACAGACCCCTCCTGCT 2.0.2**

**96 ATGGGTGTTTCCAAGAAGCTTTAGGGAT ATCCCTAAAGCTTGTCACAGACCCCTCCTGCT 2.2.2**

**97 ATGGGTGTTTCCAAGAAGCTTTAGGGAT TATCCCTAAAGCTTGTCACAGACCCCTCCTGCT 2.3.1**

**98 ATGGGTGTTTCCAAGAAGCTTTAGGGA TTATCCCTAAAGCTTGTCACAGACCCCTCCTGCT 3.4.0**

**99 ATGGGTGTTTCCAAGAAGCTTTAGGG ATCCCTAAAGCTTGTCACAGACCCCTCCTGCT 4.2.2**

**100 ATGGGTGTTTCCAAGAAGCTTTAGGGG ATCCCTAAAGCTTGTCACAGACCCCTCCTGCT 4.3.0**

**101 ATGGGTGTTTCCAAGAAGCTTTAGGG TTATCCCTAAAGCTTGTCACAGACCCCTCCTGCT 4.4.1**

**102 ATGGGTGTTTCCAAGAAGCTTTAGG CTAAAGCTTGTCACAGACCCCTCCTGCT 7.0.0**

**103 ATGGGTGTTTCCAAGAAGCTTTAGG CTAAAGCTTGTCACAGACCCCTCCTGCT 7.0.0**

**104 ATGGGTGTTTCCAAGAAGCTT GTCACAGACCCCTCCTGCT 20.0.6**

**105 ATGGGTGTTTCCAAGAAGCTT GTCACAGACCCCTCCTGCT 20.0.6**

**106 ATGGGTGTTTCCAAGAAGCTT GTCACAGACCCCTCCTGCT 20.0.6**

**107 ATGGGTGTTTCCAAGAAGCTT GTCACAGACCCCTCCTGCT 20.0.6**

**108 ATGGGTGTTTCCAAGAAGCTT GTCACAGACCCCTCCTGCT 20.0.6**

**109 ATGGGTGTTTCCAAGAAGCTTTAGACCCTCN TGCT 32.7.5**

**110 TAG GGC 179.0.1**

**111 CATT AGCTT 393.0.4**

**112 CATTCCCG AGCTT 393.4.5**

**113 CATTCCCG AGCTT 393.4.5**

**114 CATTCCCGGTT AGCTT 393.7.5**

**115 TTT ATA 394.0.0**

**116 CATCC TAAAG 397.0.2**

**117 ACAG TCAC 459.0.1**

**118 AAGA ACC 521.0.1**

**119 AAGA AAC 521.0.2**

**120 TACT TCTG 659.0.2**

**Young Kidney Fibroblasts**

**ATGGGTGTTTCCAAGAAGCTTTAGGGATAA CCCTAAAGCTTGTCACAGACCCCTCCTGCT cut ends**

**121 ATGGGTGTTTCCAAGAAGCTTTAGGGATAA TCCCTAAAGCTTGTCACAGACCCCTCCTGCT 0.1.1**

**122 ATGGGTGTTTCCAAGAAGCTTTAGGGATAA TCCCTAAAGCTTGTCACAGACCCCTCCTGCT 0.1.1**

**123 ATGGGTGTTTCCAAGAAGCTTTAGGGATA TCCCTAAAGCTTGTCACAGACCCCTCCTGCT 1.1.2**

**124 ATGGGTGTTTCCAAGAAGCTTTAGGGATA TCCCTAAAGCTTGTCACAGACCCCTCCTGCT 1.1.2**

**125 ATGGGTGTTTCCAAGAAGCTTTAGGGATA TCCCTAAAGCTTGTCACAGACCCCTCCTGCT 1.1.2**

**126 ATGGGTGTTTCCAAGAAGCTTTAGGGATA TCCCTAAAGCTTGTCACAGACCCCTCCTGCT 1.1.2**

**127 ATGGGTGTTTCCAAGAAGCTTTAGGGATAAC CCTAAAGCTTGTCACAGACCCCTCCTGCT 1.1.4**

**128 ATGGGTGTTTCCAAGAAGCTTTAGGGATN ATCCCTAAAGCTTGTCACAGACCCCTCCTGCT 1.3.2**

**129 ATGGGTGTTTCCAAGAAGCTTTAGGGAT ATCCCTAAAGCTTGTCACAGACCCCTCCTGCT 2.2.2**

**130 ATGGGTGTTTCCAAGAAGCTTTAGGGAT TATCCCTAAAGCTTGTCACAGACCCCTCCTGCT 2.3.1**

**131 ATGGGTGTTTCCAAGAAGCTTTAGGGAT TATCCCTAAAGCTTGTCACAGACCCCTCCTGCT 2.3.2**

**132 ATGGGTGTTTCCAAGAAGCTTTAGGG TTATCCCTAAAGCTTGTCACAGACCCCTCCTGCT 4.4.1**

**133 ATGGGTGTTTCCAAGAAGCTTTAGG GTTATCCCTAAAGCTTGTCACAGACCCCTCCTGCT 5.5.4**

**134 ATGGGTGTTTCCAAGAAGCTTTAGG GTTATCCCTAAAGCTTGTCACAGACCCCTCCTGCT 5.5.4**

**135 ATGGGTGTTTCCAAGAAGCTT GTCACAGACCCCTCCTGCT 20.0.6**

**136 ATGGGTGTTTCCAAGAAGCTT GTCACAGACCCCTCCTGCT 20.0.6**

**137 ATGGGTGTTTCCAAGAAG GAGCTTGTCACAGACCCCTCCTGCT 20.1.5**

**138 ATGGGTGTTTCCA(38) TGTCACAGACCCCTCCTGCT 27.38.0**

**139 ATGGGTGTTTCCAAGAA CAGACCCCTCCTGCT 28.0.0**

**140 ATGGGTGTTTCCAAGAAGCTTTAGGGATAA (138)CCC 46.138.3**

**141 ATT TCACAGACCCCTCCTGCT 47.0.3**

**142 TGC TTTGC 297.0.5**

**143 TGC TTTGC 297.0.5**

**144 TTTGCAAACTTC AGA 297.7.5**

**145 TTTCA GGTC 365.0.0**

**146 ATGGGTGTTTCCAAGAAGCTTTAGGGATAA (123)CTC 414.123.2**

**147 AAGA AAC 521.0.2**

**148 CCC TCCATCTTGCCC 644.0.12**

**149 CCC TCCATCTTGCCC 644.0.12**

**150 CCCT (31)ACT 672.31.0**

**Old Kidney Fibroblasts**

**ATGGGTGTTTCCAAGAAGCTTTAGGGATAA CCCTAAAGCTTGTCACAGACCCCTCCTGCT cut ends**

**151 ATGGGTGTTTCCAAGAAGCTTTAGGGATAA TCCCTAAAGCTTGTCACAGACCCCTCCTGCT 0.1.1**

**152 ATGGGTGTTTCCAAGAAGCTTTAGGGATA TCCCTAAAGCTTGTCACAGACCCCTCCTGCT 1.1.2**

**153 ATGGGTGTTTCCAAGAAGCTTTAGGGATAAC CCTAAAGCTTGTCACAGACCCCTCCTGCT 1.1.4**

**154 ATGGGTGTTTCCAAGAAGCTTTAGGGATAAC CCTAAAGCTTGTCACAGACCCCTCCTGCT 1.1.4**

**155 ATGGGTGTTTCCAAGAAGCTTTAGGGAT CCCTAAAGCTTGTCACAGACCCCTCCTGCT 2.0.2**

**156 ATGGGTGTTTCCAAGAAGCTTTAGGGAT CCCTAAAGCTTGTCACAGACCCCTCCTGCT 2.0.2**

**157 ATGGGTGTTTCCAAGAAGCTTTAGGGAT TATCCCTAAAGCTTGTCACAGACCCCTCCTGCT 2.3.1**

**158 ATGGGTGTTTCCAAGAAGCTTTAGGG CCTAAAGCTTGTCACAGACCCCTCCTGCT 3.0.0**

**159 ATGGGTGTTTCCAAGAAGCTTTAGGG CCCTAAAGCTTGTCACAGACCCCTCCTGCT 4.0.0**

**160 ATGGGTGTTTCCAAGAAGCTTTAGGG TCCCTAAAGCTTGTCACAGACCCCTCCTGCT 4.1.0**

**161 ATGGGTGTTTCCAAGAAGCTTTAGGG NATCCCTAAAGCTTGTCACAGACCCCTCCTGCT 4.3.0**

**162 ATGGGTGTTTCCAAGAAGCTTTAGGG TTATCCCTAAAGCTTGTCACAGACCCCTCCTGCT 4.4.4**

**163 ATGGGTGTTTCCAAGAAGC(11) CCCTAAAGCTTGTCACAGACCCCTCCTGCT 11.11.0**

**164 ATGGGTGTTTCCAAGAAGCTTTAGGG TCACAGACCCCTCCTGCT 16.0.1**

**165 ATGGGTGTTTCCAAGAAGCTT GTCACAGACCCCTCCTGCT 20.0.6**

**166 ATGGGTGTTTCCAAGAAGCTT GTCACAGACCCCTCCTGCT 20.0.6**

**167 AAAGA AATG 114.0.3**

**168 ATGGGTGTTTCCAAGAAGCTTTAGGG CTC 121.0.0**

**169 CCCT GTTATCCCTAAAGCTTGTCACAGACCCCTCCTGCT 191.5.4**

**170 TGC TTTGC 297.0.5**

**171 CTA GTG 317.0.1**

**172 AAGG TCA 341.0.1**

**173 CTA ATGGAAGG 348.7.2**

**174 TCAA GTC 365.0.0**

**175 CTG AAG(11)TG 387.0.16**

**176 GGG ACCT 486.0.0**

**177 AAGA ACC 521.0.1**

**178 AAGA AAC 521.0.2**

**179 GGGGG TCAA 646.0.3**

**180 GTG AAG 746.0.0**

**Young Lung Fibroblasts**

**ATGGGTGTTTCCAAGAAGCTTTAGGGATAA CCCTAAAGCTTGTCACAGACCCCTCCTGCT cut ends**

**181 ATGGGTGTTTCCAAGAAGCTTTAGGGATA ATCCCTAAAGCTTGTCACAGACCCCTCCTGCT 1.2.1**

**182 ATGGGTGTTTCCAAGAAGCTTTAGGGAT TATCCCTAAAGCTTGTCACAGACCCCTCCTGCT 2.3.1**

**183 ATGGGTGTTTCCAAGAAGCTTTAGGGAT CCCTAAAGCTTGTCACAGACCCCTCCTGCT 2.0.2**

**184 ATGGGTGTTTCCAAGAAGCTTTAGG GTTATCCCTAAAGCTTGTCACAGACCCCTCCTGCT 5.5.4**

**185 ATGGGTGTTTCCAAGAAGCTTTAG TCCCTAAAGCTTGTCACAGACCCCTCCTGCT 6.1.0**

**186 ATGGGTGTTTCCAAGAAGCTTTA TCCCTAAAGCTTGTCACAGACCCCTCCTGCT 7.1.3**

**187 ATGGGTGTTTCCAAGAAGCTTTT ATCCCTAAAGCTTGTCACAGACCCCTCCTGCT 7.3.2**

**188 ATGGGTGTTTCCAAGAAGCTTT CCCTAAAGCTTGTCACAGACCCCTCCTGCT 8.0.1**

**189 ATGGGTGTTTCCAAGA (35)TGTCACAGACCCCTCCTGCT 10.35.0**

**190 ATGGGTGTTTCCAAGACGC(29) GCGTGTCACAGACCCCTCCTGCT 18.31.2**

**191 ATGGGTGTTTCCAAGAAGCTT GTCACAGACCCCTCCTGCT 20.0.6**

**192 ATGGGTGTTTCCAAGAAGCTT GTCACAGACCCCTCCTGCT 20.0.6**

**193 ATGGGTGTTTCCAAGAAGCTT GTCACAGACCCCTCCTGCT 20.0.6**

**194 ATGGGTGTTTCCAAGAAGCTT GTCACAGACCCCTCCTGCT 20.0.6**

**195 ATGGGTGTTTCCAAGAAGCTT GTCACAGACCCCTCCTGCT 20.0.6**

**196 ATGGGTGTTTCCAAGAAGCTT GTCACAGACCCCTCCTGCT 20.0.6**

**197 ATGGGTGTTTCCAAGAAGCTT (17)CACCCCTCCTGCT 20.18.1**

**198 ATGGGTGTTTCCAAGACGCG(31) TGTCACAGACCCCTCCTGCT 24.35.4**

**199 ATGGGTGTTTCCAAGACGCG(31) TGTCACAGACCCCTCCTGCT 24.35.4**

**200 ATGGGTGTTTCC GACAGGCT 25.5.0**

**201 ATGGGTG(43) TGTCACAGACCCCTCCTGCT 33.43.2**

**202 ATGGGTGTTTCCAAGAAGCTTTAGGGATA (59)CTT 60.59.1**

**203 CCCCTC (49)TTGTCACAGACCCCTCCTGCT 115.51.6**

**204 AAG CAA 273.0.0**

**205 ATATTT GACC 424.0.0**

**206 ATAT CTGACC 426.2.0**

**207 CTT AAAC 455.0.2**

**208 CAT CCA 520.0.0**

**209 GAAG AGAC 805.2.2**

**210 GAAG AGAC 805.2.2**

**Old Lung Fibroblasts**

**ATGGGTGTTTCCAAGAAGCTTTAGGGATAA CCCTAAAGCTTGTCACAGACCCCTCCTGCT cut ends**

**211 ATGGGTGTTTCCAAGAAGCTTTAGGGATA TCCCTAAAGCTTGTCACAGACCCCTCCTGCT 1.1.2**

**212 ATGGGTGTTTCCAAGAAGCTTTAGGGATAAC CCTAAAGCTTGTCACAGACCCCTCCTGCT 1.1.4**

**213 ATGGGTGTTTCCAAGAAGCTTTAGGGAT CCCTAAAGCTTGTCACAGACCCCTCCTGCT 2.0.2**

**214 ATGGGTGTTTCCAAGAAGCTTTAGGG CCCTAAAGCTTGTCACAGACCCCTCCTGCT 4.0.0**

**215 ATGGGTGTTTCCAAGAAGCTTTAGGG CCCTAAAGCTTGTCACAGACCCCTCCTGCT 4.0.0**

**216 ATGGGTGTTTCCAAGAAGCT CTAAAGCTTGTCACAGACCCCTCCTGCT 12.0.3**

**217 ATGGGTGTTTCCAAGACGC(29) GCGTGTCACAGACCCCTCCTGCT 18.31.2**

**218 ATGGGTGTTTCCAAGACGC(29) GCGTGTCACAGACCCCTCCTGCT 18.31.2**

**219 ATGGGTGTTTCCAAGAAGCTT GTCACAGACCCCTCCTGCT 20.0.6**

**220 ATGGGTGTTTCCAAGAAGCTT GTCACAGACCCCTCCTGCT 20.0.6**

**221 ATGGGTGTTTCCAAGAAGCTT GTCACAGACCCCTCCTGCT 20.0.6**

**222 ATGGGTGTTTCCAAGAAGCTT GTCACAGACCCCTCCTGCT 20.0.6**

**223 ATGGGTGTTTCCAAGAAGCTT GTCACAGACCCCTCCTGCT 20.0.6**

**224 ATGGGTGTTTCCAAGAAGCTT GTCACAGACCCCTCCTGCT 20.0.6**

**225 ATGGGTGTTTCCAAGAAGCTT GTCACAGACCCCTCCTGCT 20.0.6**

**226 ATGGGTGTTTCCAGG AAGCTTGTCACAGACCCCTCCTGCT 22.1.6**

**227 ATGGGTGTTTCCAAGACGCG(31) TGTCACAGACCCCTCCTGCT 24.35.4**

**228 TGGAAA AGA 396.0.5**

**229 TGGAAA AGA 396.0.5**

**230 GCCCTT GCCT 445.1.5**

**231 AAGA AAC 521.0.2**

**232 ACTT AAG 578.0.1**

**233 TAG TCCCCAGA 715.0.8**

**234 GAAG AGAC 805.2.2**

**235 TGA AGGGA 808.3.1**

**236 AAGA ACCC 809.1.3**

**237 TAAAGACCTTGCTG ACA 873.0.14**

**238 TAAAGACCTTGCTG ACA 873.0.14**

**239 TAAAGACCTTGC ACA 886.0.12**

**240 GTG CTGA 969.0.1**

**Young Skin Fibroblasts**

**ATGGGTGTTTCCAAGAAGCTTTAGGGATAA CCCTAAAGCTTGTCACAGACCCCTCCTGCT cut ends**

**241 ATGGGTGTTTCCAAGAAGCTTTAGGGATA TCCCTAAAGCTTGTCACAGACCCCTCCTGCT 1.1.2**

**242 ATGGGTGTTTCCAAGAAGCTTTAGGGATA TCCCTAAAGCTTGTCACAGACCCCTCCTGCT 1.1.2**

**243 ATGGGTGTTTCCAAGAAGCTTTAGGGATA TATCCCTAAAGCTTGTCACAGACCCCTCCTGCT 1.3.2**

**244 ATGGGTGTTTCCAAGAAGCTTTAGGGATA TATCCCTAAAGCTTGTCACAGACCCCTCCTGCT 1.3.2**

**245 ATGGGTGTTTCCAAGAAGCTTTAGGGATA TATCCCTAAAGCTTGTCACAGACCCCTCCTGCT 1.3.2**

**246 ATGGGTGTTTCCAAGAAGCTTTAGGGAT CCCTAAAGCTTGTCACAGACCCCTCCTGCT 2.0.2**

**247 ATGGGTGTTTCCAAGAAGCTTTAGGGAT CCCTAAAGCTTGTCACAGACCCCTCCTGCT 2.0.2**

**248 ATGGGTGTTTCCAAGAAGCTTTAGGGAT TATCCCTAAAGCTTGTCACAGACCCCTCCTGCT 2.3.1**

**249 ATGGGTGTTTCCAAGAAGCTTTAGGG TCCCTAAAGCTTGTCACAGACCCCTCCTGCT 4.1.0**

**250 ATGGGTGTTTCCAAGAAGCTTTAGGG ATCCCTAAAGCTTGTCACAGACCCCTCCTGCT 4.2.2**

**251 ATGGGTGTTTCCAAGAAGCTTTAGGG TATCCCTAAAGCTTGTCACAGACCCCTCCTGCT 4.3.0**

**252 ATGGGTGTTTCCAAGAAGCTTTAGGGA AAGCTTGTCACAGACCCCTCCTGCT 8.0.0**

**253 ATGGGTGTTTCCAAGAAGCTTTAGGGA AGCTTGTCACAGACCCCTCCTGCT 9.0.1**

**254 ATGGGTGTTTCCAAGAAGCTTTAGGGA AGCTTGTCACAGACCCCTCCTGCT 9.0.1**

**255 ATGGGTGTTTCCAAGAAGCTTTAGGGAT GTCACAGACCCCTCCTGCT 11.0.0**

**256 ATGGGTGTTTCCAAGAAGCTT AAAGCTTGTCACAGACCCCTCCTGCT 13.0.1**

**257 ATGGGTGTTTCCAAGAAGCTT GTCACAGACCCCTCCTGCT 20.0.6**

**258 ATGGGTGTTTCCAAGAAGCTT GTCACAGACCCCTCCTGCT 20.0.6**

**259 ATGGGTGTTTCCAAGAAGCTT GTCACAGACCCCTCCTGCT 20.0.6**

**260 ATGGGTGTTTCCAAGAAGCTT GTCACAGACCCCTCCTGCT 20.0.6**

**261 ATGGGTGTTTCCAAGAAG TCACAGACCCCTCCTGCT 23.0.1**

**262 ATGGGTGTTTCCAAGACGCG(31) TGTCACAGACCCCTCCTGCT 24.35.4**

**263 AGC AC 212.0.1**

**264 TCA GGTC 365.0.0**

**265 ATATTT GACC 424.0.0**

**266 ATAT CTGACC 426.2.0**

**267 ATAT CTGACC 426.2.0**

**268 AAGA ACC 521.0.1**

**269 AAGA ACC 521.0.1**

**270 CAG GTC 628.1.3**

**Old Skin Fibroblasts**

**ATGGGTGTTTCCAAGAAGCTTTAGGGATAA CCCTAAAGCTTGTCACAGACCCCTCCTGCT cut ends**

**271 ATGGGTGTTTCCAAGAAGCTTTAGGGATA TCCCTAAAGCTTGTCACAGACCCCTCCTGCT 1.1.2**

**272 ATGGGTGTTTCCAAGAAGCTTTAGGGATAAC CCTAAAGCTTGTCACAGACCCCTCCTGCT 1.1.3**

**273 ATGGGTGTTTCCAAGAAGCTTTAGGGAT CCCTAAAGCTTGTCACAGACCCCTCCTGCT 2.0.2**

**274 ATGGGTGTTTCCAAGAAGCTTTAGGGAT CCCTAAAGCTTGTCACAGACCCCTCCTGCT 2.0.2**

**275 ATGGGTGTTTCCAAGAAGCTTTAGGGAT CCCTAAAGCTTGTCACAGACCCCTCCTGCT 2.0.2**

**276 ATGGGTGTTTCCAAGAAGCTTTAGGGAT CCCTAAAGCTTGTCACAGACCCCTCCTGCT 2.0.2**

**277 ATGGGTGTTTCCAAGAAGCTTTAGGGAT ATCCCTAAAGCTTGTCACAGACCCCTCCTGCT 2.2.2**

**278 ATGGGTGTTTCCAAGAAGCTTTAGGGAT ATCCCTAAAGCTTGTCACAGACCCCTCCTGCT 2.2.2**

**279 ATGGGTGTTTCCAAGAAGCTTTAGGGAT ATCCCTAAAGCTTGTCACAGACCCCTCCTGCT 2.2.2**

**280 ATGGGTGTTTCCAAGAAGCTTTAGGGAT TATCCCTAAAGCTTGTCACAGACCCCTCCTGCT 2.3.1**

**281 ATGGGTGTTTCCAAGAAGCTTTAGGGA TATCCCTAAAGCTTGTCACAGACCCCTCCTGCT 3.3.2**

**282 ATGGGTGTTTCCAAGAAGCTTTAGGG CCCTAAAGCTTGTCACAGACCCCTCCTGCT 4.0.0**

**283 ATGGGTGTTTCCAAGAAGCTTTAGGG TTATCCCTAAAGCTTGTCACAGACCCCTCCTGCT 4.4.3**

**284 ATGGGTGTTTCCAAGAAGCTTTAGG GTTATCCCTAAAGCTTGTCACAGACCCCTCCTGCT 5.5.4**

**285 ATGGGTGTTTCCAAGAAGCTTTAGGGAT AAGCTTGTCACAGACCCCTCCTGCT 7.0.2**

**286 ATGGGTGTTTCCAAGAAGCTTTA TCCCTAAAGCTTGTCACAGACCCCTCCTGCT 7.1.3**

**287 ATGGGTGTTTCCAAGAAGCTT GTCACAGACCCCTCCTGCT 20.0.6**

**288 ATGGGTGTTTCCAAGAAGCTT GTCACAGACCCCTCCTGCT 20.0.6**

**289 ATGGGTGTTTCCAAGACGCG(31) TGTCACAGACCCCTCCTGCT 24.35.4**

**290 ATGGGTGTTTCC TGGT 154.0.3**

**291 GGT GTT 247.1.2**

**292 TCCCG TCCAC 379.0.2**

**293 TACAGAGG TAG 770.0.8**

**294 TACAGAGG GTC 778.0.8**

**295 GAAG AGAC 805.2.2**

**296 GCG CTC 835.0.0**

**297 GCG CTC 835.0.0**

**298 GCG TC 836.0.0**

**299 TGT TAC 913.0.1**

**300 GCTA TTT 929.0.0**

^a^ I-SceI recognition sequence is nonpalindromic, therefore two inverted I-SceI sites in the reporter construct generate incompatible DNA overhangs.

^b^**D.I.M.**: (Deletion, Insertion and Microhomology repeat) sequences in bp. In each sequence, underlined nucleotides denote Insertions and red nucleotides denote Microhomology repeats.
